# Supplementary material for: A Critical View of the Application of the APEX Software (Aqueous Photochemistry of Environmentally-Occurring Xenobiotics) to Predict Photoreaction Kinetics in Surface Freshwaters
Source: Molecules. 2019 Dec 18;25(1):9. doi: 10.3390/molecules25010009 (PMC7017383; doi:10.3390/molecules25010009)
Supplement: Supplementary file 1 [file molecules-25-00009-s001.zip › 3.2.4_gcc-4.4.0.zip!/3.2.4_gcc-4.4.0/3.2.4_gcc-4.4.0/doc/octave/html/interpreter/Demonstration-Functions.html]

Demonstration Functions - Untitled


Previous: Test Functions,
Up: Test and Demo Functions


---

### B.2 Demonstration Functions

— Function File: **demo** ('name',n)  
> Runs any examples associated with the function 'name'.
> Examples are stored in the script file, or in a file with the same
> name but no extension somewhere on your path. To keep them separate
> from the usual script code, all lines are prefixed by `%!`. Each
> example is introduced by the keyword 'demo' flush left to the prefix,
> with no intervening spaces. The remainder of the example can contain
> arbitrary Octave code. For example:
>
> ```
>              %!demo
>              %! t=0:0.01:2*pi; x = sin(t);
>              %! plot(t,x)
>              %! %-------------------------------------------------
>              %! % the figure window shows one cycle of a sine wave
> ```
>
> Note that the code is displayed before it is executed, so a simple
> comment at the end suffices. It is generally not necessary to use
> disp or printf within the demo.
>
> Demos are run in a function environment with no access to external
> variables. This means that all demos in your function must use
> separate initialization code. Alternatively, you can combine your
> demos into one huge demo, with the code:
>
> ```
>              %! input("Press <enter> to continue: ","s");
> ```
>
> between the sections, but this is discouraged. Other techniques
> include using multiple plots by saying figure between each, or
> using subplot to put multiple plots in the same window.
>
> Also, since demo evaluates inside a function context, you cannot
> define new functions inside a demo. Instead you will have to
> use `eval(example('function',n))` to see them. Because eval only
> evaluates one line, or one statement if the statement crosses
> multiple lines, you must wrap your demo in "if 1 <demo stuff> endif"
> with the 'if' on the same line as 'demo'. For example,
>
> ```
>             %!demo if 1
>             %!  function y=f(x)
>             %!    y=x;
>             %!  endfunction
>             %!  f(3)
>             %! endif
> ```
>
>
>
>
> **See also:** test, example.

— Function File: **rundemos** (directory)

— Function File: **example** ('name',n)  
— Function File: [x, idx] = **example** ('name',n)  
> Display the code for example n associated with the function
> 'name', but do not run it. If n is not given, all examples
> are displayed.
>
> Called with output arguments, the examples are returned in the form of
> a string x, with idx indicating the ending position of the
> various examples.
>
> See `demo` for a complete explanation.
>
>
>
> **See also:** demo, test.

— Function File: **speed** (f, init, max\_n, f2, tol)  
— Function File: [order, n, T\_f, T\_f2] = **speed** (...)  
> Determine the execution time of an expression for various n.
> The n are log-spaced from 1 to max\_n. For each n,
> an initialization expression is computed to create whatever data
> are needed for the test. If a second expression is given, the
> execution times of the two expressions will be compared. Called
> without output arguments the results are presented graphically.
>
> f: The expression to evaluate. max\_n: The maximum test length to run. Default value is 100. Alternatively, use `[min_n,max_n]` or for complete control, `[n1,n2,...,nk]`. init: Initialization expression for function argument values. Use k for the test number and n for the size of the test. This should compute values for all variables listed in args. Note that init will be evaluated first for k = 0, so things which are constant throughout the test can be computed then. The default value is x `= randn (`n`, 1);`. f2: An alternative expression to evaluate, so the speed of the two can be compared. Default is `[]`. tol: If tol is `Inf`, then no comparison will be made between the results of expression f and expression f2. Otherwise, expression f should produce a value v and expression f2 should produce a value v2, and these shall be compared using `assert(`v`,`v2`,`tol`)`. If tol is positive, the tolerance is assumed to be absolute. If tol is negative, the tolerance is assumed to be relative. The default is `eps`. order: The time complexity of the expression `O(a n^p)`. This is a structure with fields `a` and `p`. n: The values n for which the expression was calculated and the execution time was greater than zero. T\_f: The nonzero execution times recorded for the expression f in seconds. T\_f2: The nonzero execution times recorded for the expression f2 in seconds. If it is needed, the mean time ratio is just `mean(T_f./T_f2)`.
>
> The slope of the execution time graph shows the approximate
> power of the asymptotic running time `O(n^p)`. This
> power is plotted for the region over which it is approximated
> (the latter half of the graph). The estimated power is not
> very accurate, but should be sufficient to determine the
> general order of your algorithm. It should indicate if for
> example your implementation is unexpectedly `O(n^2)`
> rather than `O(n)` because it extends a vector each
> time through the loop rather than preallocating one which is
> big enough. For example, in the current version of Octave,
> the following is not the expected `O(n)`:
>
> ```
>           speed ("for i = 1:n, y{i} = x(i); end", "", [1000,10000])
> ```
>
> but it is if you preallocate the cell array `y`:
>
> ```
>           speed ("for i = 1:n, y{i} = x(i); end", ...
>                  "x = rand (n, 1); y = cell (size (x));", [1000, 10000])
> ```
>
> An attempt is made to approximate the cost of the individual
> operations, but it is wildly inaccurate. You can improve the
> stability somewhat by doing more work for each `n`. For
> example:
>
> ```
>           speed ("airy(x)", "x = rand (n, 10)", [10000, 100000])
> ```
>
> When comparing a new and original expression, the line on the
> speedup ratio graph should be larger than 1 if the new expression
> is faster. Better algorithms have a shallow slope. Generally,
> vectorizing an algorithm will not change the slope of the execution
> time graph, but it will shift it relative to the original. For
> example:
>
> ```
>           speed ("v = sum (x)", "", [10000, 100000], ...
>                  "v = 0; for i = 1:length (x), v += x(i); end")
> ```
>
> A more complex example, if you had an original version of `xcorr`
> using for loops and another version using an FFT, you could compare the
> run speed for various lags as follows, or for a fixed lag with varying
> vector lengths as follows:
>
> ```
>           speed ("v = xcorr (x, n)", "x = rand (128, 1);", 100,
>                  "v2 = xcorr_orig (x, n)", -100*eps)
>           speed ("v = xcorr (x, 15)", "x = rand (20+n, 1);", 100,
>                  "v2 = xcorr_orig (x, n)", -100*eps)
> ```
>
> Assuming one of the two versions is in xcorr\_orig, this
> would compare their speed and their output values. Note that the
> FFT version is not exact, so we specify an acceptable tolerance on
> the comparison `100*eps`, and the errors should be computed
> relatively, as `abs((`x `-` y`)./`y`)` rather than
> absolutely as `abs(`x `-` y`)`.
>
> Type `example('speed')` to see some real examples. Note for
> obscure reasons, you can't run examples 1 and 2 directly using
> `demo('speed')`. Instead use, `eval(example('speed',1))`
> and `eval(example('speed',2))`.
